# Predicting probable Alzheimer’s disease using linguistic deficits and biomarkers

**DOI:** 10.1186/s12859-016-1456-0

**Published:** 2017-01-14

**Authors:** Sylvester O. Orimaye, Jojo S-M. Wong, Karen J. Golden, Chee P. Wong, Ireneous N. Soyiri

**Affiliations:** 1 Intelligent Health Research Group, School of Information Technology, Monash University, Jalan Lagoon Selatan, Bandar Sunway, 47500 Malaysia; 2 Jeffrey Cheah School of Medicine and Health Sciences, Monash University, Jalan Lagoon Selatan, Bandar Sunway, 47500 Malaysia; 3 Centre for Medical Informatics, Usher Institute for Population Health Sciences & Informatics, The University of Edinburgh, Teviot Place, Edinburgh, EH8 9AG UK

**Keywords:** Alzheimer’s disease, Neurolinguistics, Clinical diagnostics, Prediction, Machine learning

## Abstract

**Background:**

The manual diagnosis of neurodegenerative disorders such as Alzheimer’s disease (AD) and related Dementias has been a challenge. Currently, these disorders are diagnosed using specific clinical diagnostic criteria and neuropsychological examinations. The use of several Machine Learning algorithms to build automated diagnostic models using low-level linguistic features resulting from verbal utterances could aid diagnosis of patients with probable AD from a large population. For this purpose, we developed different Machine Learning models on the DementiaBank language transcript clinical dataset, consisting of 99 patients with probable AD and 99 healthy controls.

**Results:**

Our models learned several syntactic, lexical, and *n*-gram linguistic biomarkers to distinguish the probable AD group from the healthy group. In contrast to the healthy group, we found that the probable AD patients had significantly less usage of syntactic components and significantly higher usage of lexical components in their language. Also, we observed a significant difference in the use of *n*-grams as the healthy group were able to identify and make sense of more objects in their *n*-grams than the probable AD group. As such, our best diagnostic model significantly distinguished the probable AD group from the healthy elderly group with a better Area Under the Receiving Operating Characteristics Curve (AUC) using the Support Vector Machines (SVM).

**Conclusions:**

Experimental and statistical evaluations suggest that using ML algorithms for learning linguistic biomarkers from the verbal utterances of elderly individuals could help the clinical diagnosis of probable AD. We emphasise that the best ML model for predicting the disease group combines significant syntactic, lexical and top *n*-gram features. However, there is a need to train the diagnostic models on larger datasets, which could lead to a better AUC and clinical diagnosis of probable AD.

**Electronic supplementary material:**

The online version of this article (doi:10.1186/s12859-016-1456-0) contains supplementary material, which is available to authorized users.

## Background

Alzheimer’s disease (AD) is the most common form of dementia [1–4]. However, the manual diagnosis of AD and other types of dementia is currently challenging [5–8]. Moreover, AD has been typically diagnosed through extensive neuropsychological examinations using a series of cognitive tests containing a set of questions and images [9–12]. For example, the Mini-Mental State Examination (MMSE) and the Montreal Cognitive Assessment (MoCA) screening tools are composed of a series of questions and cognitive tests that assess different cognitive abilities [13]. With a maximum score of 30, an MMSE score of 27 and above is suggestive of not having a dementia related disease [4]. The challenge with these cognitive tests is that they are administered manually. Also, the accuracy of the tests depends on the clinician’s level of experience and their ability to diagnose different sub-types of the disease [14, 15]. Often, researchers and clinicians need to combine other cognitive tests with the MMSE [15, 16]. In most cases, a reasonably long interval of up to two years is necessary to use the MMSE to distinguish between the sub-types of dementia; for example, from Mild Cognitive Impairment (MCI) to AD [4, 17]. As the dementia subtypes include probable and possible AD, Vascular Dementia, Dementia with Lewy Bodies (DLB), Mixed Dementia, Parkinson’s Disease and others^1^, it is challenging for the MMSE to manually distinguish effectively between all these possible categories over a large population [16].

Research has also shown that the reliability of the neuropsychological examinations for diagnosing AD and related dementias could be limited. For example, the National Institute on Aging and the Alzheimer’s Association workgroups on diagnostic guidelines for Alzheimer’s disease has called for effective methods (other than the usual neuropsychological measures through mental status examination) that could be used to diagnose AD and related dementia [18, 19]. As part of the recommendations in [19], an effective diagnostic measure should include amnestic and nonamnestic presentations that are able to capture cognitive deficits from retelling a recently observed scenario, language presentation (lexical, syntactic, and others), visuospatial presentation of objects and their semantic interpretations, and executive function such as reasoning with a sense of judgment in accomplishing a specific task.

As opposed to the ad hoc use of neuropsychological examinations, linguistic ability captured from verbal utterances could be a good indication of symptoms of AD and other dementia related diseases [20]. The premise is that neurodegenerative disorders (ND) deteriorate nerve cells that control cognitive, speech and language processes [21], which consequentially translates to how patients compose verbal utterances [22]. According to [23], syntactic processing in acquired language disorders such as Aphasia in adults has shown promising findings, encouraging further study in identifying effective syntactic techniques. Similarly, [24] emphasised the significance of lexical-semantic components of a language, part of which is observable during utterance acquisition at a younger age. Locke’s work further highlighted that as the lexical capacity increases, syntactic processing becomes automated, thence leading to changes in language. As such, it was inferred that the effects of a specific language disorder can cause changes to the lexical and syntactic processes governing language and verbal utterances.

In [3], the efficacy of using complex syntactic features to classify MCI – which is a precursor to AD – was demonstrated. That study is relevant, as it used spoken language characteristics to discriminate between 37 patients with MCI and 37 in the healthy elderly group. In that work, a total of 21 linguistic features from speech and syntactic measures including pause and syntactic annotations were extracted. Seven linguistic features were found to be statistically significant for immediate logic memory. A combination of several test scores and those linguistic features achieved up to 86.1% AUC. On the other hand, our current work distinguishes patients with probable AD using several low-level syntactic and lexical features, which are more representative of the language space of both the disease and the healthy controls. In this work, we also introduced for the first time an extensive use of word *n*-grams to detect patients with probable AD with up to 1000 useful and discriminating features.

Similarly, [25] investigated the significance of lexical and syntactic features from the verbal narratives of AD patients by performing several statistical tests based on 121 elderly participants comprising 60 subjects with AD and 61 healthy subjects. Their lexical features were composed of word-finding difficulties, immediate word repetition of isolated words, word revisions, semantic substitutions, and phonemic paraphasias. For syntactic features, coordinated sentences, subordinated sentences, and reduced sentences were examined. Upon performing and making comparisons between the parametric Student’s *t*-test (*t*) and the non-parametric Mann-Whitney test (U), only word-finding difficulties, immediate repetitions, word revisions, coordinated sentences, and reduced sentences were found to be statistically significant with *p* = 0.001 at the 95% Confidence Interval (CI). Further posthoc analysis with the Wald test (Wald *X* ^2^) showed that immediate word repetitions, word revisions, and coordinated sentences could be used to distinguish those patients with AD from the healthy elderly group. While [25] did not perform any evaluation using Machine Learning (ML) algorithms, we focus on the feasibility of effectively distinguishing the patients with probable AD from the healthy elderly group by learning additional syntactic, lexical, and *n*-gram features with an effective ML algorithm.

More recently, [26] proposed a machine learning technique to classify patients with primary progressive Aphasia from connected speech. In that work, distinguishing syntactic and semantic features were identified from three different groups, which included patients with semantic dementia (SD), progressive nonfluent Aphasia (PNFA), and healthy controls. Unlike the healthy controls, high usage of nouns were found to characterise the SD group, while high usage of verbs characterises the PNFA group. In contrast, our study focuses on distinguishing between the patients with probable AD from the healthy controls. Also, our study includes the use of syntactic, lexical, and *n*-gram features for building diagnostic models.

As such, in this paper, we investigate an effective computational diagnostic model for predicting probable AD from verbal utterances. A potential clinical usefulness of this work is the ability to predict the probable AD phenotype, which might have surpassed the MCI stage. More importantly, there is a reasonable interval in the pathological pathway between the prodromal AD and full-blown AD or Dementia [27, 28]. There is also growing evidence for the likelihood of predicting the AD pathological process in the brain even before clinical symptoms emanates [28, 29]. As such, it is still important to detect the probable AD phenotype from the elderly population. We believe that the detection of probable AD could help early intervention before progressing to the full-blown AD or Dementia where symptoms become substantially pronounced [28]. In addition, our work proposed a simple yet effective technique to automate the diagnosis of the disease on a large-scale using speech transcripts only. We see this as part of the efforts to actualize an automated telediagnostic tool for the remote diagnosis or screening of the disease from a large population where the manual administration of the neuropsychological examinations could be limited. Also, the diagnostic model could help do a probable assessment of patients with the disease for possible rehabilitation of aspects of their language (cognitive) dysfunction and necessary recommendation of social support that could decrease the caregiver’s burden [30, 31].

We use several linguistic features from the transcribed verbal utterances produced by 99 healthy control individuals and 99 probable AD patients from the DementiaBank dataset^2^. We proposed the diagnostic models based on the effective Artificial Intelligence (AI) techniques in Natural Language Processing (NLP), combined with a reliable ML algorithm that learns several low-level linguistic features and identifies the probable AD group from the healthy elderly group. The computational models proposed in this paper use three essential components of language. First, we explore the lexical information contained in the vocabulary space of the patients with AD [32, 33]. Second, we use the syntactic representation and constituents to understand the variations in the complexity of grammar usage between the healthy and disease groups [32, 34]. Finally, we introduce the *n*-gram model [35], which captures the pattern of the sequence of words in the language of the disease and healthy elderly groups. We emphasise that the machine learning approach has proven to be effective in clinical diagnostics [3, 12, 36, 37]. Our predictive models achieved a better Area Under the ROC Curve (AUC) in distinguishing the probable AD group from the healthy elderly group.

## Methods

It is common in clinical research to conduct an investigation on the actual patients (or subjects). However, previous research studies have made available a series of clinical datasets that reduce the investigation time considerably. Although this study did not involve direct interaction with actual patients, we retrospectively focused on understanding the linguistic patterns from the verbal utterances found of existing patients. Earlier, we have discussed those utterances to be present in the transcription files contained in the DementiaBank dataset, and we will describe the dataset further in the dataset section. In this paper, our focus was to use extensive syntactic, lexical, and *n*-gram features for building the diagnostic models. As such, we identified a total of the top 1000 features for the probable AD and healthy elderly groups in our dataset. The features combine syntactic, lexical, and *n*-gram features from a possible 16,926 feature space. 9 of those features are syntactic, 14 are lexical features, 1 is a confounding factor (age), and the remaining 976 are explicit word *n*-grams derived from the vocabulary space of both probable AD and healthy elderly groups. We will describe the features in detail later in this section. We performed statistical tests on the extracted features. Both the Student’s *t*-test (*t*) and the Mann-Whitney test (U) were performed and followed by Multiple Logistic Regression (MLR) that showed the most significant features. The final ML models were built using a reliable learning algorithm, which we will discuss later. We compared our technique with [3, 25, 37] as benchmark papers.

### Datasets

In this study, an existing DementiaBank clinical dataset was used. The dataset resulted from a longitudinal study on Alzheimer’s disease and related Dementia, conducted by the University of Pittsburgh School of Medicine, which was funded by the National Institute on Aging^3^. The dataset contains transcripts of verbal interviews of patients with probable and possible AD, MCI, and other related dementia. Participants responded to the interview in the English language based on the description of the Cookie-Theft picture component, which is part of the Boston Diagnostic Aphasia Examination (BDAE) [38]. Note that the BDAE Cookie-Theft picture has been shown to be clinically relevant in identifying linguistic deficits in both Alzheimer’s disease and Aphasia patients [39]. Although language transcripts from other descriptions are available as part of the AphasiaBank^4^ corpus (e.g. the descriptive Broken Window picture and the narrative Cinderella fairy tale picture), those transcripts are specifically for patients with Aphasia.

During the interview, patients were given the Cookie-Theft picture and were told to discuss everything they could see happening in the picture. The patients’ verbal utterances were recorded and then transcribed into the CHAT transcription format [40]. The CHAT transcription format^5^ is the result of a set of computational tools developed to expedite the automated transcription of audio data for research purposes. This format is used commonly in child language research as part of the Child Language Data Exchange System (CHILDES) [40], which is a component of the TalkBank^6^ project. In this study, we extract the transcribed patient sentences from the CHAT files and then pre-process the sentences for feature extraction. Although some demographic details are available in the DementiaBank dataset, we have selected only *age*, in addition to the extracted features, in order to measure the significance of the disease with respect to age.

### Participants

The DementiaBank dataset categorised the participants into Dementia, Control, and Unknown groups. The Dementia group consists of 169 probable and possible AD patients. The AD patients have an approximate age range of 49 to 90 years. On the other hand, the Control group consists of 99 healthy elderly individuals without any reported diagnosis and with an approximate age range of 46 to 81 years. Since our study focuses on the binary diagnosis of patients with probable AD from the healthy elderly group, we formed the AD group with the first 99 probable AD patients, equal to the number of healthy control individual available in the dataset. Note that the probable AD patients correspond to patients with a probable diagnosis of Alzheimer’s disease. Thus, in our experiments, we will use the 99 probable AD patients to discriminate the 99 healthy control individuals. It is inferred that using the patients with probable AD could improve the sensitivity of our model to correctly predict surfacing linguistic deficits that may eventually lead to full-blown AD or Dementia.

Finally, it is important to mention that the longitudinal study that was conducted on the participants in the DementiaBank dataset includes multiple visits at different time intervals, some as far apart as one to two years. As such, in our study, the selected 99 subjects are based on the transcript files from the last visit to each of the participants in the probable AD and Control groups. For the purpose of this paper, we will subsequently refer to the probable AD group as PrADG and the healthy elderly control group as HEG.

### Feature extraction

Several features were extracted from the transcript files. First, we extracted every CHAT symbol in the transcript files and stored them according to their frequencies and positions in each sentence. We emphasise that some CHAT symbols represent both explicit and implicit features that describe the lexical capability of each patient. For example, having the CHAT symbol [//] at a specific position within a sentence implies that the patient was retracing a verbal error, which precedes that position, and at the same time attempting to make a correction. Similarly, the CHAT symbol [/] indicates immediate word repetition [40]. On the other hand, it is non-trivial to extract the syntactic features without performing syntactic parsing on the sentences. As such, using the Stanford Parser [41], we generated the syntactic tree structure of each sentence and extracted features as appropriate.

### Syntactic features

We investigated a number of features that are seen to demand complex syntactic processing, including the three syntactic features (*coordinated*, *subordinated*, and *reduced* sentences) evaluated by [25] and the *dependency distance* feature evaluated in [3, 42]. Again, all syntactic features are extracted from the syntactic tree structures produced by the Stanford Parser. The proposed syntactic features are as follows:

- **Coordinated sentences**: Coordinated sentences are those whose clauses are combined using coordinating conjunctions. The number of occurrences for this feature per patient narrative is obtained based on the frequency of the coordinating conjunction Part-Of-Speech (POS) tag (CC) detected in the parse tree structure.
- **Subordinated sentences**: Subordinated sentences are those that are subordinate to the independent primary sentence to which they are linked. Similarly, the number of occurrence for this feature per patient narrative is obtained based on the frequency of the sub-sentences indicated by the POS tag (S) detected in the parse tree structure.
- **Reduced sentences**: Following the definition set out by [25], this feature represents those subordinated sentences without a conjunction but with nominal verb forms (which are either participles or gerund). To obtain the count for this feature, the frequencies of POS tags (VBG and VBN) are used.
- **Number of predicates**: The number of predicates found in every patient’s narrative can be seen as another estimation of the sentence complexity. The predicates are extracted using a rule-based algorithm, which locates transitive verbs that are followed by one or more arguments. We emphasise that the importance of predicate-argument structures has been explored in the literature for text classification tasks [43, 44].
- **Average number of predicates**: The average number of predicates per patient narrative is investigated to study its effect.
- **Dependency distance**: This feature was used in the study of [42] as a way to measure grammatical complexity in patients with Alzheimer’s disease. The distance value is calculated based on the sum of all the dependency distances, in which each dependency distance is the absolute difference between the serial position of two words that participate in a dependency relation.
- **Number of dependencies**: For a purpose similar as to the syntactic dependency distance, the number of unique syntactic dependency relations found in every patient’s narrative is examined.
- **Average dependencies per sentence**: We also consider the average number of the unique dependency relations per sentence.
- **Production rules**: Production rules derived from parse trees have been explored in a number of NLP related classification tasks [45, 46]. We investigate this feature by counting the number of unique production rules in the context-free grammar form extracted from each patient’s narrative.

### Lexical features

The lexical features used in this study include the *revision* and *repetition* features proposed in [47] and evaluated in [25]. The remaining features include the lexical features that show better improvement with our models.

- **Utterances**: The total number of utterances per patient was computed. Each utterance is identified to start from the beginning of verbal communication to the next verbal pause length, such as punctuation or a CHAT symbol that represents a specific break in communication [48]. A sentence could have one or more utterances and an utterance could be one word, a phrase or a clause. It has been identified that utterance acquisitions create the grammatical lexicon for a language [24]. Thus, we hypothesise that the absolute number of utterances in a conversation could show the linguistic strength of a potential patient.
- **Mean Length of Utterances (MLU)**: We measure the structural organisation of sentences using the MLU. The MLU is the ratio of the total number of words to the number of utterances [48]. MLU has been specifically used to measure grammar growth in children with Specific Language Impairment (SLI) [49]. In this study, we investigate the significance of MLU in determining language disorder in patients with AD.
- **Function words**: We compute the total number of function words in the patient’s narrative. Function words coordinate the meaning of a sentence, and they are essential attributes to brain and language processing [50].
- **Unique words**: We measure the total number of unique words as the absolute word count minus the number of immediately repeated words.
- **Word count**: We estimated the total word count including repeated words.
- **Character length**: We measure the absolute character length of the patient’s narrative.
- **Total sentences**: This is the complete number of sentences in the patient’s description.
- **Repetitions**: This is the number of immediate word repetitions in the patient’s narrative [25, 47].
- **Revisions**: This feature estimates the count of pause positions where the patient retraced a preceding error and then made a correction [25, 40, 47].
- **Morphemes**: To capture the morphology structure of the patient’s narrative, we measured the number of morphemes. Each morpheme represents a word or a part of it that cannot be further divided [51].
- **Trailing off indicator**: we captured the number of instances at which a patient trails off before completing an utterance or a sentence. The trailing off indicator is part of the CHAT symbols [40].
- **Word replacement**: we identified instances where a patient used incorrect word or phrase in an utterance and makes an attempt to reuse the right word or phrase. The word replacement indicator is part of the CHAT symbols [40].
- **Incomplete words**: there are instances where a patient did not produce all the syllables or letters of a word. For example, a patient might say just *goi* in *going*. The non-completion of a word indicator is part of the CHAT symbols [40].
- **Filler words**: individual words that are attached to the CHAT fusion marker symbol are identified as filler words. The words appear in the form of *uh* or *ehm*. The fusion marker is part of the CHAT symbols [40].

### N-gram features - bigrams and trigrams

The use of word *n*-grams is popular in NLP especially for developing language models that can characterise the lexical usage in the grammar contained in a dataset [32, 52]. A word *n*-gram is the sequence of words identified as an independent representation of a part of the grammar of an utterance or a sentence [52]. *n*, in this case, represents the number of words in the sequence. For instance, when *n* is 1, it is called a unigram, which has only one word. Similarly, a bigram and a trigram have *n* equal to 2 and 3 respectively, and it is not uncommon to use higher order *n*-grams (i.e. *n*≥3) for clinical machine learning tasks [32]. For the purpose of this study, our *n*-grams consist of bigram and trigram features only, which are from the transcripts of both the disease and control groups. We put emphasis on bigrams and trigrams because both feature types have been known to have performed with reasonable accuracy in other NLP and ML tasks [53]. Since *n*-gram features can be enormous, we will only evaluate their contributions for distinguishing the PrADG from the HEG during our ML experiments.

## Results and discussion

### Statistical evaluation of syntactic and lexical features

One of the challenges that we encountered in evaluating the features above is that some features are just not very evenly distributed. An exception to that is the confounding feature *age*. For age, it is our assumption that the DementiaBank study was designed to cover normally distributed participants regarding age range. For the other generated features, it is understandable, since each patient would give specific attributes that show the severity of the disease over time. As such, we performed one parametric test (Student’s *t*-test (*t*)) and one non-parametric test (Mann-Whitney test (U)) and then compared the results of the two tests [25]. We used a 95% confidence interval (CI) for both lower and upper bounds, and a *p*<0.05 showed statistical significance. All tests performed are two-tailed using the IBM Statistical Package for the Social Sciences (SPSS) version 20.0.0^7^. The parametric and non-parametric tests achieved similar 2-tailed significance results in both cases as shown in Table 1. We therefore chose the parametric results for further statistical evaluation.

**Table 1:** Statistical analysis of syntactic and lexical features from the PrADG and HEG based on Student’s *t*-test

	PrADG	HEG	*t*	df	*p*	95%
	MEAN(SD)	MEAN(SD)				CI(Difference)
Confounding feature						
Age	70.45(8.916)	65.26(8.388)	3.621	148	<0.000*	2.36 to 8.01
Syntactic features						
Coordinated sentences	5.09(3.22)	4.85(2.99)	0.55	196	0.584	-0.63 to 1.11
Subordinated sentences	5.42(3.63)	5.13(3.19)	0.60	196	0.547	-0.66 to 1.25
Reduced sentences	2.95(2.48)	4.08(2.57)	-3.15	196	0.002*	-1.84 to -0.42
Number of Predicates	5.54 (3.44)	6.94(3.53)	-2.83	196	0.005*	-2.38 to -0.43
Avr. predicates per sentence	0.42(0.19)	0.58(0.22)	-5.48	196	<0.000*	-0.22 to -0.10
Number of dependencies	100.90(53.36)	100.81(51.44)	0.01	196	0.990	-14.60 to 14.78
Avr.dependency per sentence	8.21(2.69)	8.78(2.36)	-1.58	196	0.115	-1.28 to 0.14
Dependency distance	16.21(7.75)	17.09(7.05)	-0.83	196	0.405	-2.95 to 1.197
Production rules	128.61(52.00)	126.75(46.35)	0.26	196	0.791	-11.95 to 15.67
Lexical features						
Utterances	50.52(35.61)	31.05(15.49)	4.99	196	<0.000*	11.77 to 27.16
MLU	2.65(1.70)	4.03(2.25)	-4.86	196	<0.000*	-1.94 to -0.82
Function words	58.00(35.84)	59.71(35.33)	-0.34	196	0.736	-11.68 to 8.27
Unique words	115.92(63.96)	116.03(59.92)	-0.01	196	0.990	-17.48 to 17.26
Word count	127.79(72.62)	127.69(68.45)	0.01	196	0.992	-19.68 to 19.88
Character length	562.35(313.33)	583.21(316.65)	-0.47	196	0.642	-109.15 to 67.44
Total sentences	14.01(8.33)	12.48(5.56)	1.52	196	0.131	-0.46 to 3.51
Repetitions	2.09(3.08)	0.70(1.03)	4.27	196	<0.000*	0.75 to 2.04
Revision	4.54(5.27)	2.02(2.20)	4.38	196	<0.000*	1.38 to 3.65
Number of morphemes	117.35(76.56)	117.09(69.65)	0.02	196	0.980	-20.25 to 20.78
Trailing off	0.85(1.18)	0.14(0.38)	5.67	196	<0.000*	0.46 to 0.95
Word replacement	1.28(1.37)	0.44(0.77)	5.30	196	<0.000*	0.53 to 1.15
Incomplete words	5.56(4.05)	3.11(3.42)	4.59	196	<0.000*	1.39 to 3.49
Filler words	6.47(6.89)	4.30(3.53)	2.79	196	0.006*	0.64 to 3.71

*PrADG Probable Alzheimer’s Disease Group (N=99), *HEG* Healthy Elderly Group (N=99), *SD* Standard Deviation, *df* degree of freedom, *CI* Confidence Interval

Our analysis showed that the statistically significant syntactic features of the PrADG have lower means compared to the HEG. Our observation is that the probable AD group appear to have difficulties in constructing complex sentences, unlike the control group. We suggest that effective use of predicates and reduced structures could be of vital importance to appropriately measure the linguistic capability in patients with probable AD. On the other hand, statistically significant lexical features of the PrADG have higher means compared to the HEG, except for MLU with just 1.38 difference (PrADG=2.65; HEG=4.03; *p*<0.000). This result makes sense, for example, the PrADG performed more immediate word repetitions and made more revisions on grammatical errors in their narrative. The PrADG has a higher number of utterances because of pauses and syntactic errors. In many cases, we observed that the PrADG retraced their errors, which led to more utterances. The PrADG also tended to express themselves for a longer duration in their narrative, leading to an increase in the number of sentences. We suggest that a higher number of the significant lexical features could help distinguish the patients with probable AD from the healthy group.

We then conducted a post hoc test using the MLR analysis on the resulting significant features from the *t*-test. The analysis further demonstrated the distinguishing strength of the features to predict the disease groups from the healthy elderly group. Because age is a common factor attributed with AD and the PrADG is on average five years older than the HEG (PrADG=70.45; HEG=65.26; *p*<0.000), we adjusted for the effect of age as a confounding feature in the MLR analysis. We present the results of the analysis using the Wald test (Wald *X* ^2^) and the Odds Ratio (OR) or *Exp*(*B*) at the 95% CI as shown in Table 2.

**Table 2:** Multiple logistic regression analysis on confounding, syntactic, and lexical features from the PrADG and HEG

Features	*β*	S.E	Wald *X* ^2^	*p*	OR	95% CI or OR
Age	-0.095	0.030	9.70	0.002*	0.91	0.86 to 0.96
Reduced Sentences	0.185	0.083	5.02	0.025*	1.20	1.02 to 1.41
MLU	0.300	0.142	4.49	0.034*	1.35	1.02 to 1.78
Trailing off	-1.300	0.437	8.85	0.003*	0.27	0.12 to 0.64
Constant	2.319	2.108	1.21	0.271	10.17	-

*PrADG(N=99); HEG(N=99); *S.E* Standard Error, *OR* Odds Ratio or Exp(*β*), *CI* Confidence Interval

The OR emphasises the likelihood of having probable AD when the description contains the distinguishing features. Lower *β* values decrease the probability of having probable AD. In Table 2, we see that there were significant 20% (OR=1.20, *p* = 0.025) odds that *reduced sentences* would appear in the picture descriptions of patients with probable AD compared to the healthy controls. Similarly, we observed significant 35% (OR=1.35, *p* = 0.034) odds that MLU would predict the PrADG compared to the HEG. On the other hand, the odds that *age* and *Trailing off* would predict the PrADG significantly reduced by 9% (OR=0.91, *p* = 0.002) and 73% (OR=0.27, *p* = 0.003). This evaluation leaves out the *number of predicates*, *average predicate per sentence*, *utterances*, *repetitions*, *revisions*, *word replacement*, *incomplete words*, and *filler words*. Interestingly, *repetitions* were found to be a significant distinguishing feature in [25], albeit on 121 patients. In our case, we assume that repeated words would be less significant given the small data sample while the absolute count of predicates in a discourse (not at the sentence level) could be more representative of the groups instead of their average per sentence. We will compare the ML predictive performance of our significant features to [3, 25], and also [37] which is a precursor to this study.

### Evaluations with ML algorithm

We built different feature models and performed different sets of the experiment to verify the hypothesis that automatic diagnostic models can predict probable AD with reasonable performance (Additional file 1). We developed our models with the Sequential Multiple Optimisation (SMO), which is a variant of the Support Vector Machines (SVM) algorithm ([54]). As such, the SMO implementation of the SVM in the Waikato Environment for Knowledge Analysis (WEKA) Java API^8^ was used in our experiments [55]. Hence, we will refer to that implementation as SVM.

As shown in Table 3, we identified the optimal kernel and hyperparameters for tuning the SVM on a separate development set by using Auto-Weka [56] with the top combined 1000 features. Since the DementiaBank dataset contains multiple visits to the participants, we used the transcript files from the second to the last visit to create the development set from which we identified the hyperparameters. That set consists of random 40 transcript files from the patients with probable AD and random 40 transcript files from the healthy controls. Note that the development set is not a subset of the actual PrADG and HEG data used for training and testing our model. On the other hand, the training and testing data consists of transcript files from the last visit only (i.e. 99 probable AD and 99 Control). The interval between the two visits is at least one year. We will evaluate our predictive models on the last visit PrADG and HEG data only.

**Table 3:** Best hyperparameters found for SVM on PrADG/HEG validation dataset (PrADG=40;HEG=40) with Auto-Weka

Algorithm	Seed	Training time	Optimisation method	Hyperparameters
SVM-top-1000-	2	3 hours	SMAC	-C 1.4786727172414378 -N 1 -K "RBFKernel -G
PrADG/HEG				0.0014243946679106075”

seed = random integer for randomising the data during training; SMAC is a Bayesian optimisation method proposed as part of Auto-Weka

We measured the performance of our ML models with the ROC [3, 57, 58]. The area under the ROC curve, known as the AUC, is commonly used for evaluating the performance of clinical diagnostic and predictive models [59]. The AUC makes a tradeoff between the sensitivity (true positive rate) and the specificity (true negative rate) [60–62]. The sensitivity is the percentage of positive instances which were accurately classified as positive. On the other hand, the specificity computes the percentage of negative instances which were accurately classified as negative. In evaluating a classifier, the ROC curve plots the sensitivity against 1-specificity (false positive rate) [3, 58, 59]. That curve shows the growth of the classification threshold from the very positive threshold where every instance is classified as positive to the very negative threshold where every instance is classified as negative. When the sensitivity of a classifier is 0.0 and the specificity is 1.0, then the confidence score of the classifier is below the set threshold. Conversely, when the specificity is 0.0 and sensitivity is 1.0, it means the confidence score of the classifier is above the set threshold. A random classifier has an AUC of 0.5 with a diagonal line connecting the origin (0, 0) to the final point (1, 1). An AUC of 1.0 starts the ROC curve from (0, 0) to (1, 0), hence a perfect classifier [59], which ranks all positive instances above all negative instances. Note that the AUC is the equivalent of the Wilcoxon-Mann-Whitney statistic [63], which shows that a classifier is likely to rank randomly selected positive cases higher than randomly selected negative cases. While different clinical diagnostic scenarios make different tradeoff with the AUC, an AUC that is greater than 0.75 is usually recommended for clinical purposes [3, 58, 64].

To conduct an informed comparison with the findings from our baselines, we estimated the AUC using the leave-pair-out cross-validation (LPOCV), which produces an unbiased estimate of the AUC, especially for clinical diagnostics [3, 58]. Choosing LPOCV as a reliable clinical evaluation technique has also been extensively argued and justified in the literature [65, 66]. Unlike other cross-validation techniques, every pair of positive and negative cases are evaluated on a model trained on the rest of the cases. For example, since our dataset consists of 99 PrADG subjects and 99 HEG subjects, each round of the LPOCV selects a unique pair of one PrADG and one HEG subjects as the test set for evaluating a model trained with the remaining 98 PrADG and 98 HEG subjects. The evaluation score is the classifier’s confidence *c*, computed for each example in the example test pair, and can be used to calculate the Wilcoxon-Mann-Whitney statistic using the AUC implementation^9^ in the WEKA Java API. The AUC is defined as follows:

1 $$ c(p,n) =\left\{ \begin{array}{ll} 1 &\text{if}~c(p) > c(n) \\ 0 & \text{otherwise} \end{array} \right.  $$

2 $$ AUC(c,P,N) = \frac{1} {|P||N|}\sum\limits_{p \in P} \sum\limits_{n \in N} c(p,n)  $$

where *c*(*e*) is the classifier’s confidence score for an example *e*, *P* is a set of positive (PrADG) examples, and *N* is a set of negative (HEG) examples. We also compute the variance of the AUC and then report the standard deviation [3, 58]. The variance $\sigma ^{2}_{AUC}$ is computed as follows, where *A* denotes the AUC:

3 $$ {{\begin{aligned} {}\sigma^{2}_{AUC} = \frac{A(1-A) + (|P|-1)\left(\frac{A}{2-A} - A^{2}\right) + (|N| - 1)\left(\frac{2A^{2}}{1+A} - A^{2}\right)} {|P||N|} \end{aligned}}}  $$

### Baselines

We performed our evaluation in comparison with the significant features identified in the three related papers [3, 25, 37]. We have implemented the 3 significant features proposed in [25] as part of our model, which are *coordinated sentences*, *revision*, and *repetition*. In [3], the Wechsler Logical Memory task [67], was used to collect language and speech data from a narrative memory task, which required the subjects to listen to a story and then recall everything they can from the story. That procedure allowed the subjects to formulate original language structures on their own. The task also helps to capture both linguistic and memory deficiencies from the participants by using various language and speech measures. As such, we implemented all the 7 Wechsler Logical Memory I significant features from [3], which include Words per clause, Part-Of-Speech cross-entropy, content density, Standard Pause Rate, Total Phonation Time, Phonation Rate, and Transformed Phonation Rate. Note that these features were extracted from our dataset (Additional file 2) and not from the dataset used in [3].

As discussed in the Datasets section, it is worth mentioning that the language and speech data from the Cookie-Theft picture description task upon which we built our models was collected differently from the Wechsler Logical Memory task. Nevertheless, the Cookie-Theft picture description task required the subjects to formulate their language structures by describing the Cookie-Theft scenes in no particular order. This process is quite important in diagnosing patients with AD as many linguistic defects will likely show in the inability of the patients to describe the scenes meaningfully. Also, we did not consider the Wechsler Logical Memory II significant features as a baseline, because that task captures much longer memory deficiencies by making the subjects recall the story after 30 minutes or more. Our goal was to detect immediate linguistic deficiencies from the patients, which could aid quick diagnostics rather than delay them. Finally, additional lexical and *n*-gram features have been used to extend the significant features in [37].

### Diagnostic task

Aside from the 23 syntactic and lexical features discussed earlier, our dataset generated 16,903 *n*-gram features. Altogether, we generated 16,926 features. As such, we combined all syntactic, lexical, and *n*-gram features, and used the Information Gain (IG) [68] together with the *Ranker* algorithm in WEKA to select the top ranked combined 1000 features (*top-combined-1000*) upon which we built our diagnostic model. We believe that feature selection is essential to building our model as it leads to overall better performance compared to using all possible features with many redundancies [68]. Thus, using the LPOCV, we performed experiments to determine the performance of the *top-combined-1000* features. Also, we evaluated an additional eight other different feature groupings, among which are the 23 syntactic and lexical features (*23-syntactic-lexical-only*), *top-1000-n-gram-only* features, and the three baselines’ features (i.e. [3, 25, 37]). As shown in Table 4, our model gave a better AUC with the *top-combined-1000* (AUC=0.93; s.d.=1.89) and the *top-1000-n-gram-only* (AUC=0.91; s.d.=2.14) features, where s.d. denotes standard deviation. More importantly, most of our proposed features performed better than the three baselines implemented in this study.

**Table 4:** Classification AUC and standard deviation comparison between the proposed and baseline features on the PrADG/HEG

Models	AUC(s.d.)
23-syntactic-lexical-only	**0.80(3.12)**
23-syntactic-lexical-Roark-7	**0.82(2.98)**
11-t-test-syntactic-lexical-sig.	**0.81(3.05)**
3-MLR-syntactic-lexical-sig.	0.70(3.71)
top-combined-1000	**0.93(1.89)**
top-1000-n-gram-only	**0.91(2.14)**
Orimaye-5-baseline	0.75(3.43)
Delira-3-baseline	0.54(4.06)
Roark-7-baseline	0.73(3.53)

23-syntactic-lexical-only = proposed syntactic and lexical features; 23-syntactic-lexical-Roark-7 = proposed syntactic and lexical features combined with Roark’s Wechsler Logical Memory I 7 significant features; 3-MLR-syntactic-lexical-sig. = MLR significant features; 11-t-test-syntactic-lexical-sig. = t-test significant features; top-combined-1000 = top ranked 1000 features consisting of syntactic, lexical, and n-gram features; top-1000-n-gram-only = top 1000 bigrams and trigrams without syntactic and lexical features

Boldfaced means better results

In comparison to the word alignment features in [58], our *top-combined-1000* and *top-1000-n-gram-only* models showed better AUC, although those authors did not use the same set of probable AD patients in our dataset. The alignment features include *graph-based content word summary score* (AUC=0.70; s.d.=3.2); *graph-based content word word-level score* (AUC=0.82; s.d.=2.6); *Berkeley content word summary score* (AUC=0.68; s.d.=3.3); *Berkeley content word word-level score* (AUC=0.83; s.d.=2.5); *BLEU* (AUC=0.70; s.d.=3.2); and *Unigram precision* (AUC=0.63; s.d.=3.4). Unlike [58], we did not limit the participants’ descriptions to a certain number of words.

The performance of the 23 syntactic and lexical features (AUC=0.80; s.d.=3.12) demonstrates their vital roles in identifying the linguistic biomarkers in patients with probable AD. We see that the result supports the statistical analysis presented in Table 1. We observed significant differences between the means of the features from the PrADG and HEG statistically. The model with *t*-test significant features results in a significantly better AUC than all our baselines, and the model with just three MLR significant features (AUC=0.70; s.d.=3.71) gives a better AUC than the three significant features in the [25] baseline. Note that we did not consider confounding feature *age* as part of the statistical significant features to the SVM because confounding features are known to introduce bias to AUC [62]. Significantly, we recorded a better AUC by combining the 23 syntactic and lexical features with the significant features from [3] (AUC=0.82; s.d.=2.98). We see this improvement as a positive indication that our model has the potential to be improved with further significant features.

We see that the *top-combined-1000* and the *top-1000-n-gram-only* features are the particular strength of our models because of their statistical significance on the DementiaBank dataset. Unlike some other lexical features, the *n*-gram features do not require any manual annotation and can be easily collected on any other clinically recommended picture in the same manner as the Cookie Theft picture for the purpose of training a predictive model. We also believe that the *n*-gram features captured most of the important linguistic distinctions between the PrADG and the HEG.

Following the statistical evaluation performed for the proposed syntactic and lexical features (Table 1), we performed the *t*-test for the top ranked 20 *n*-gram features for both PrADG and HEG to show the importance of *n*-grams to the diagnostic task. Note that we generated the top 20 n-grams in the same way as the top 1000 n-grams. As shown in Table 5, all the top ranked 20 *n*-gram features show significant *p*-values at the 95% CI. It is also of note that most of the *n*-grams consist of recognisable object names from the Cookie-Theft picture. For example, *the window*, *the mother is*, *the stool*, *the sink*, and *cookie out of*. More importantly, the PrADG mostly had lower means for each of the *n*-grams compared to the HEG with mostly higher means. This result is consistent with the study conducted by [69], which suggests that the HEG were able to identify and make sense of more objects than the PrADG.

**Table 5:** Statistical analysis of the top 20 *n*-gram features from the PrADG and HEG based on Student’s *t*-test

*n*-gram	PrADG MEAN(SD)	HEG MEAN(SD)	*t*	df	*p*	95% CI(Difference)
the window	0.12(0.38)	0.62(0.82)	-5.45	196	<0.000*	-0.67 to -0.32
mother is	0.15(0.44)	0.60(0.70)	-5.37	196	<0.000*	-0.61 to -0.28
be quiet	0.01(0.10)	0.22(0.44)	-4.66	196	<0.000*	-0.30 to -0.12
is open	0.04 (0.24)	0.31(0.58)	-4.29	196	<0.000*	-0.40 to -0.15
the mother	0.22(0.46)	0.61(0.74)	-4.37	196	<0.000*	-0.56 to -0.21
tipping over	0.00(0.00)	0.17(0.43)	-3.98	196	<0.000*	-0.26 to -0.09
window is	0.01(0.10)	0.19(0.40)	-4.43	196	<0.000*	-0.26 to -0.10
girl is	0.14(0.40)	0.44(0.57)	-4.29	196	<0.000*	-0.44 to -0.16
is tipping	0.00(0.00)	0.14(0.35)	-4.02	196	<0.000*	-0.21 to -0.07
the window is	0.01(0.10)	0.18(0.39)	-4.27	196	<0.000*	-0.25 to -0.09
the mother is	0.11(0.35)	0.42(0.61)	-4.45	196	<0.000*	-0.45 to -0.17
of the cookie	0.08(0.31)	0.32(0.49)	-4.16	196	<0.000*	-0.36 to -0.13
the stool	0.33(0.74)	0.58(0.67)	-2.41	196	0.017*	-0.44 to -0.04
is overflowing	0.05(0.33)	0.22(0.42)	-3.20	196	0.002*	-0.28 to -0.07
the sink	0.68(0.91)	1.17(1.01)	-3.62	196	<0.000*	-0.76 to -0.22
this is	0.25(0.61)	0.02(0.14)	3.68	196	<0.000*	0.11 to 0.36
cookie out	0.00(0.00)	0.11(0.32)	-3.50	196	0.001*	-0.17 to -0.05
cookie out of	0.00(0.00)	0.11(0.32)	-3.50	196	0.001*	-0.17 to -0.05
a cookie out	0.00(0.00)	0.10(0.30)	-3.32	196	0.001*	-0.16 to -0.04
off the cookie	0.01(0.10)	0.14(0.35)	-3.59	196	<0.000*	-0.20 to -0.06

*PrADG Probable Alzheimer’s Disease Group (N=99), *HEG* Healthy Elderly Group (N=99), *SD* Standard Deviation, *df* degree of freedom, *CI* Confidence Interval

Finally, we performed the MLR analysis to adjust for the effect of age on the top-20 n-gram features. The purpose is to show whether age affects the predictive power of the *n*-grams. As shown in Table 6, we see that the odds that age would predict probable AD significantly reduced by 14%. On the other hand, features such as *the window*, *the mother*, and *girl is* had statistically significant odds ratios (OR) of 7.00 (*p*<0.000), 4.6 (*p*=0.024), and 6.7 (*p*<0.000) for predicting patients with probable AD compared to the healthy controls. As such, we believe that the lexical, syntactic, and *n*-gram features have the potential to predict patients with probable AD with minimal effects from age.

**Table 6:** Multiple logistic regression analysis on confounding and n-gram features from the PrADG and HEG

Features	*β*	S.E	Wald *X* ^2^	*p*	OR	95% CI of OR
Age	-0.152	0.040	14.56	<0.000*	0.86	0.79 to 0.93
the window	1.946	0.519	14.08	<0.000*	7.00	2.53 to 19.35
the mother	1.526	0.674	5.13	0.024*	4.60	1.23 to 17.23
be quiet	4.263	1.299	10.77	0.001*	71.00	5.57 to 905.46
girl is	1.905	0.541	12.40	0.000*	6.72	2.33 to 19.39
this is	-2.967	1.256	5.58	0.018*	0.05	0.00 to 0.60
Constant	7.742	2.512	9.50	0.002*	2302.57	-

*PrADG(N=99); HEG(N=99); *SE* Standard Error, *OR* Odds Ratio or Exp(*β*), *CI* Confidence Interval

### Limitations

A limitation of this study could be the limited size of the datasets, which is often a challenge in clinical research. We believe that an increase in the data sample is likely to improve the performance of our proposed models for predicting probable AD from the healthy controls.

The choice of the development set for the Machine Learning algorithm is also a limitation. In this study, we used forty random transcript files from “the second to the last visit" in the DementiaBank dataset to find the hyperparameters for the SVM algorithm. Although the longitudinal study has at least a year interval between different visits to the patients, combined with the possible further deterioration in the cognitive speech of the patients, it is likely, that the development set forms part of the training and testing data.

Another limitation of this study is the use of CHAT symbols for the identification of some of the lexical features. Although the CHAT transcription format has been effective for analysing speech data [70], it is still not universally used for speech transcription. Also, currently, speech transcripts are manually annotated with the CHAT symbols by a carefully trained personnel [40]. As such, the practical use of some of the lexical features would require that speech transcripts from potential patients are annotated manually before using the proposed model. Note that a successful transcription is required to follow the conventions described in the CHAT Manual [40]. Nevertheless, our *top-1000-n-gram-only* features are also as useful as the *top-combined-1000* features and could be better with sufficient data sample. Moreover, the automatic AD transcript annotation with transcript symbols are potential areas for future research.

Finally, the use of the top performing *n*-gram features in this study is confined to the description of the Cookie-Theft picture. This limitation is understandable since the objects within the scenes dictate the specific *n*-gram features in the language space of the PrADG and HEG. Unless a picture with similar objects in the Cookie-Theft picture is used for collecting the speech transcript, the use of any other image with different objects is likely to generate a different set of *n*-gram features.

## Conclusions

The results of our ML experiments and statistical evaluations suggest that using ML algorithms for learning syntactic, lexical, and *n*-gram features from the verbal utterances of elderly people could help the diagnosis of probable Alzheimer’s disease. The outcome of our evaluations has verified the efforts of our baseline papers [3, 25, 58]. However, our study identified more characteristic and representative linguistic features compared to the benchmark papers. Furthermore, in comparison to [58], which performed a non-linguistic reference task with word alignment features on the same DementiaBank dataset with different number of AD patients, our *n*-gram models showed better AUC without reducing the number of words in the language transcripts of the participants. Following the results of our experiments, we emphasise that the best ML model for predicting the probable AD group combines significant syntactic, lexical and top bigram and trigram (*n*-grams) features.

Although the proposed diagnostic model has some evident limitations, we have found that it is capable of capturing cognitive deficits and/or biomarkers from amnestic and nonamnestic presentations by verbally describing the clinically relevant Cookie-Theft picture as shown by the results of our statistical and diagnostic evaluations. We anticipate that our model has the potential for positive societal impact to contribute to actualizing an automated telediagnostic tool for the remote diagnosis or screening of probable Alzheimer’s disease from a large population. Moreover, the diagnostic model could facilitate the probable assessment of patients with the disease for possible rehabilitation of aspects of their language dysfunction and necessary recommendation of social support that could decrease economic and caregiver’s burden. We plan to evaluate our models against the MMSE and MoCA diagnostic thresholds on actual AD patients in a developing country. There is also a need to train the diagnostic models on larger datasets, which could lead to a better AUC. Additionally, longitudinal studies are recommended to improve sample sizes and follow the course of the diseases over time.

## Endnotes

^1^ http://www.alz.org/dementia/types-of-dementia.asp

^2^ http://talkbank.org/DementiaBank/

^3^ http://www.nia.nih.gov/

^4^ http://talkbank.org/APhasiaBank/

^5^ http://childes.psy.cmu.edu/manuals/CHAT.pdf

^6^ http://talkbank.org/

^7^ http://www-01.ibm.com/software/analytics/spss/

^8^ http://www.cs.waikato.ac.nz/ml/weka/

^9^ https://weka.wikispaces.com/Area+under+the+curve

## Additional files

Additional file 1 Machine Learning files for all the models presented in our results, including baseline models. These files contain the transformed linguistic features from the DementiaBank dataset for both disease and control groups combined. The files appear in the WEKA Attribute-Relation File Format (ARFF). (ZIP 78.5 kb)

Additional file 2 Raw transformed data. These files contain the transformed linguistic features from the DementiaBank dataset and appear in the Comma Separated Values file format. (ZIP 18.4 kb)

## Abbreviations

AD: Alzheimer’s disease
PrADG: Probable Alzheimer’s disease group
AI: Artificial intelligence
API: Application programming interface
AUC: Area under the receiver operating characteristics curve
CHAT: Codes for the human analysis of transcripts
CI: Confidence interval
DLB: Dementia with Lewy bodies
HEG: Healthy elderly group
LPOCV: Leave-pair-out cross-validation
MCI: Mild cognitive impairment
ML: Machine learning
MLR: Multiple logistic regression
MMSE: Mini-mental state examination
MoCA: Montreal cognitive assessment
ND: Neurodegenerative disorders
NLP: Natural language processing
PNFA: Progressive NonFluent Aphasia
ROC: Receiver operating characteristics
SD: Semantic dementia
SLI: Specific language impairment
SMO: Sequential multiple optimization
SPSS: Statistical package for the social sciences
SVM: Support vector machines
WEKA: Waikato environment for knowledge analysis
